# Radiotherapy in nodal oligorecurrent prostate cancer

**DOI:** 10.1007/s00066-021-01778-1

**Published:** 2021-04-29

**Authors:** Michael Pinkawa, Daniel M. Aebersold, Dirk Böhmer, Michael Flentje, Pirus Ghadjar, Nina-Sophie Schmidt-Hegemann, Stefan Höcht, Tobias Hölscher, Arndt-Christian Müller, Peter Niehoff, Felix Sedlmayer, Frank Wolf, Constantinos Zamboglou, Daniel Zips, Thomas Wiegel

**Affiliations:** 1 Department of Radiation Oncology, MediClin Robert Janker Klinik, Bonn, Germany; 2 Department of Radiation Oncology, Universitätsklinikum RWTH Aachen, Pauwelsstr. 30, 52074 Aachen, Germany; 3 Department of Radiation Oncology, Inselspital, University of Bern, Bern, Switzerland; 4 Department of Radiation Oncology, Charité Universitätsmedizin Berlin, Berlin, Germany; 5 Department of Radiation Oncology, Universitätsklinikum Würzburg, Würzburg, Germany; 6 Department of Radiation Oncology, University Hospital, LMU Munich, Munich, Germany; 7 Xcare Praxis für Strahlentherapie Saarlouis, Xcare Gruppe, Saarlouis, Germany; 8 Department of Radiotherapy and Radiation Oncology, University Hospital Carl Gustav Carus, Technische Universität Dresden, Dresden, Germany; 9 Department of Radiation Oncology, Eberhard Karls University, Tübingen, Germany; 10 Department of Radiation Oncology, Sana Klinikum Offenbach, Offenbach, Germany; 11 Landeskrankenhaus, Universitätsklinikum der Paracelsus Medizinischen Privatuniversität Salzburg, Salzburg, Austria; 12 Department of Radiation Oncology, Universitätsklinikum Freiburg, Freiburg, Germany; 13 Department of Radiation Oncology, Universitätsklinikum Ulm, Ulm, Germany

**Keywords:** Prostate cancer, Oligorecurrence, Metastasis-directed therapy, Radiation therapy, Androgen deprivation therapy, Stereotactic body radiotherapy, Oligmometastases, Lymph node metastases

## Abstract

**Objective:**

The current article encompasses a literature review and recommendations for radiotherapy in nodal oligorecurrent prostate cancer.

**Materials and methods:**

A literature review focused on studies comparing metastasis-directed stereotactic ablative radiotherapy (SABR) vs. external elective nodal radiotherapy (ENRT) and studies analyzing recurrence patterns after local nodal treatment was performed. The DEGRO Prostate Cancer Expert Panel discussed the results and developed treatment recommendations.

**Results:**

Metastasis-directed radiotherapy results in high local control (often > 90% within a follow-up of 1–2 years) and can be used to improve progression-free survival or defer androgen deprivation therapy (ADT) according to prospective randomized phase II data. Distant progression after involved-node SABR only occurs within a few months in the majority of patients. ENRT improves metastases-free survival rates with increased toxicity in comparison to SABR according to retrospective comparative studies. The majority of nodal recurrences after initial local treatment of pelvic nodal metastasis are detected within the true pelvis and common iliac vessels.

**Conclusion:**

ENRT with or without a boost should be preferred to SABR in pelvic nodal recurrences. In oligometastatic prostate cancer with distant (extrapelvic) nodal recurrences, SABR alone can be performed in selected cases. Application of additional systemic treatments should be based on current guidelines, with ADT as first-line treatment for hormone-sensitive prostate cancer. Only in carefully selected patients can radiotherapy be initially used without additional ADT outside of the current standard recommendations. Results of (randomized) prospective studies are needed for definitive recommendations.

## Introduction

Recent advances in diagnostic techniques as well as local and systemic treatments have improved outcomes in prostate cancer (PCa) patients [1–3]. Diagnostic procedures increasingly include magnetic resonance imaging (MRI) and positron-emission tomography/computed tomography (PET/CT), currently above all using prostate-specific membrane antigen (PSMA) for improved local and systemic staging [4]. Thus, relapses can already be detected with low prostate-specific antigen (PSA) levels, which frequently lead to the diagnosis of oligorecurrence in a small number of lymph nodes after local prostate treatment (radical prostatectomy or radiotherapy) [5]. So far, it is not known if identification and treatment of low-volume nodal recurrences improves the survival of these patients.

Following primary treatment, 20–50% of high-risk patients develop biochemical recurrences [6, 7]. Generally, patients with low-volume disease are known to have a better outcome than patients with high-volume disease [8]. Commonly, androgen deprivation therapy (ADT) is initiated in recurrent disease. However, local treatment as the sole intervention or in combination with ADT is increasingly used [7].

This is similar to intensification of treatment in non-small cell lung cancer. Two randomized phase II trials in oligometastatic non-small cell lung cancer showed improved progression-free and overall survival after stereotactic ablative radiotherapy (SABR) was added to maintenance systemic therapy [9, 10]. The SABR-COMET (Comprehensive Treatment of Oligometastases) trial reported an overall survival benefit when SABR was used in addition to standard-of-care systemic therapy across various histologies (including 16 prostate cancer patients) [11, 12].

With radiotherapy being increasingly applied in oligorecurrent and oligometastatic prostate cancer, the aim of this manuscript is to define the role of radiotherapy in nodal oligorecurrent PCa.

## Materials and methods

A review of the literature was performed. The analysis focused on studies analyzing recurrence patterns after local nodal treatment and studies comparing SABR for nodal recurrences vs. observation, or SABR (synonymous with stereotactic body radiotherapy, SBRT, or involved-field radiotherapy, IFRT) for nodal recurrences vs. elective nodal radiotherapy (ENRT). The Prostate Cancer Expert Panel of the German Society of Radiation Oncology (DEGRO) discussed the results and developed treatment recommendations on the radiotherapy volume, radiotherapy technique, and role of additional systemic therapy.

## Results

### Comparison of different local nodal treatments or observation

Two prospective, randomized, multicenter phase II trials were recently published in recurrent prostate cancer, comparing surveillance or metastasis-directed therapy (MDT) [13, 14]. In the STOMP (Surveillance or MDT for Oligometastatic Prostate Cancer Recurrence) trial, patients (*n* = 62) with up to three PET-positive metastatic lesions, including 55% lymph node (LN) metastasis in both groups, were included. MDT included SABR (30 Gy in three fractions) in most patients (*n* = 25), but also surgery (*n* = 6). At a median follow-up of 3 years, the median ADT-free survival (primary endpoint) was 13 months in the surveillance group and 21 months in the MDT group (hazard ratio, HR: 0.6; *p* = 0.11). In the intention-to-treat analysis, a significant difference was only found for nodal metastases (HR 0.4 for nodal, *p* = 0.04 vs. HR 0.75, *p* = 0.51 for non-nodal metastases) [13].

The ORIOLE (Observation vs. Stereotactic Ablative Radiation for Oligometastatic Prostate Cancer) trial also included patients (*n* = 54) with up to three metastatic lesions in conventional imaging, randomized 2:1 to SABR (19.5–48 Gy in 3–5 fractions) or observation. In 61%, metastatic lesions included only lymph nodes. Biochemical or clinical progression at 6 months occurred in 19% of patients receiving SABR and 61% undergoing observation (*p* < 0.01). Total regression of PSMA radiotracer-avid disease decreased the risk of new lesions at 6 months (16 vs. 63%; *p* < 0.01). SABR was suggested to induce a systemic immune response. The presence of high-risk mutations in circulating tumor DNA (ctDNA, detectable in 22 patients) might be associated with a worse prognosis [14].

Larger patient numbers with a longer follow-up were evaluated in a multi-institutional retrospective case–control study for PET-detected nodal oligorecurrent prostate cancer (90% limited to the pelvis). In the MDT cohort, 166 patients received salvage lymph node resection and 97 patients SABR. The standard-of-care cohort included 1816 patients and was matched 3:1 with the MDT cohort. After a median follow-up of 70 months, a significant cancer-specific survival benefit resulted for patients after MDT (98.6 vs. 95.7% 5‑year survival, *p* < 0.01) [15].

A multi-institutional retrospective study (15 different centers) compared SABR vs. ENRT. This analysis included 506 patients (SABR: 309; ENRT: 197) patients with hormone-sensitive oligorecurrent PCa defined by up to five lymph node (LN) metastases in pelvic (N1: 365) or extrapelvic (M1a: 98) nodes (N1 combined with M1a: 43). The median follow-up was 36 months (interquartile range 23–56 months). SABR was defined as a minimum of 5 Gy per fraction with a maximum of 10 fractions. ENRT was defined as a minimum dose of 45 Gy in up to 25 fractions to the elective nodes (without specific definition), with or without a simultaneous integrated boost to the suspicious node(s). Nodal recurrences were detected by PET/CT in 97% (choline: *n* = 428; PSMA: *n* = 46; fluorodeoxyglucose: *n* = 17) or conventional imaging in 3% (MRI: *n* = 5; CT: *n* = 10) of cases. The 3‑year metastasis-free survival was 68% for SABR and 77% for ENRT (*p* = 0.01). Overall, ENRT was associated with significantly fewer nodal recurrences compared to SABR (20 vs. 42%; *p* < 0.001), especially with fewer pelvic recurrences (2 vs. 18%). In multivariate analysis, patients with a single LN recurrence had longer metastasis-free survival after ENRT (hazard ratio: 0.5; *p* = 0.009). Late toxicity was higher after ENRT compared to SABR (18 vs. 6%, *p* < 0.01, including 2.5% vs. 0 grade 3 or 4 toxicity). Limitations of this retrospective study include higher use of ADT (applied at the discretion of the treating physician) in the ENRT cohort and nonstandardized follow-up [16].

In a further, overall smaller retrospective study comparing ENRT (including a boost to the involved nodes) with SABR, 62 patients were included. A 3-year failure-free survival of 88 vs. 55% (*p* < 0.001) was reported by Lépinoy et al. [17]. In contrast to the multi-institutional study, ENRT was well defined in this study. ENRT included the whole pelvis as defined by the radiation therapy oncology group (RTOG; [18]). In patients with PET-positive common iliac or lower paraaortic lymph nodes, the clinical target volume (CTV) was extended up to the L2/L3 space. When lumboaortic lymph nodes were involved, the CTV was extended up to the renal arteries.

### Recurrence patterns after local nodal treatment

In an analysis of 72 patients after SABR for up to three LN recurrences (overall 89 LN; N1/M1a), 68% relapses occurred again in nodal regions. Relapses after pelvic nodal SABR (*n* = 36) were located in the pelvis (39%), retroperitoneum (3%), pelvis and retroperitoneum (22%), or in non-nodal regions (36%) [19].

De Bruycker et al. evaluated 158 LN recurrences in 82 patients with up to five LN (N1/M1a) after primary lymphadenectomy (*n* = 12) or ENRT (*n* = 12) or combined lymphadenectomy with ENRT (*n* = 56; missing information in 2 patients). In 49% of patients, recurrences were exclusively located in the true pelvis, followed by the common iliac LN (10%), retroperitoneal/inguinal LN (10%), or a combination (31%). In contrast to ENRT, limited or standard LN dissection was considered insufficient as a salvage approach. Limiting the upper border to the top of L4 instead of L5/S1 would increase lesion coverage from 43 to 67% [20].

Soldatov et al. analyzed recurrence patterns in 108 patients after ^68^Ga-prostate specific membrane antigen (PSMA) ligand PET/CT-guided RT (without additional systemic treatment) for recurrent oligometastatic disease, including in 45% pelvic and 18% extrapelvic lymph node metastases. Treatment also included regional pelvic irradiation with conventional fractionation. A total of 97% showed an initial decrease in PSA levels after RT. Recurrent disease was localized in 33 of 36 patients in a new PET/CT, 88% outside of the initial RT field, with a median distant disease-free survival of 11 months. A shift in the pattern of metastases towards more distant lymph nodes and skeletal involvement was reported. Recurrences after initially treated pelvic lymph nodes occurred most frequently in pelvic lymph nodes or the retroperitoneum [5].

## Discussion

Currently available diagnostic methods, especially ^68^Ga-PSMA PET/CT, allow the detection of oligometastatic or oligorecurrent prostate cancer even at low PSA levels. Research in metastasis-directed radiotherapy has recently gained interest with the aim of improving survival outcomes or deferring systemic treatment. To date, two prospective randomized phase II studies and several cohort studies have been published.

Two RT concepts are currently applied: SABR and ENRT. In patients with lymph node metastases, many centers opted for focal SABR. Results supporting this approach with excellent local control rates > 85% have been published by several groups [5, 13, 14]. Overall, a wide variety of fractionation concepts are used, including conventional fractionation for ENRT, moderate hypofractionation with fraction doses of 2.5–3 Gy, or ultra-hypofractionation with fraction doses of 5–10 Gy (common understanding of SABR) [21]. In two prospective phase II randomized studies, improvements of progression-free survival, ADT-free survival, and a decreased risk of new lesions were demonstrated [13, 14]. Improved cancer-specific survival was observed in a retrospective multi-institutional case–control study after a longer median follow-up of 36 months [15].

However, recurrence pattern analysis found a considerably higher percentage of recurrences close to the irradiated area following SABR in comparison to ENRT [5, 19, 20]. Thus, regional control rates and metastases-free survival could be improved with ENRT. Overall, comparative studies suggest a prognostic advantage of ENRT in comparison to SABR [16, 17]. In anatomical subregions containing a single metastatic lymph node, the minimal short diameter of tumor deposits required to reach a detection rate of 50 and 90% in ^68^Ga-PSMA PET/CT was estimated to be ≥ 2.3 and ≥ 4.5 mm, respectively [22]. Following salvage lymph node dissection, Jilg et al. [22] found the majority of false-negative subregions (13/16) in regions neighboring true-positive subregions.

In an analysis of 2694 patients treated with prostate +/− seminal vesicle radiotherapy without nodal treatment, 156 patients had their first radiographically confirmed (CT: 117; MRI: 27; PET/CT: 12) failure within the abdominopelvic lymph nodes in the context of biochemical failure [23]. Isolated failures within the pelvic nodes were detected in 60 patients and the common iliac station was involved in 55% (*n* = 33) of these patients. Extending the field to cover the common iliac stations from L5/S1 to L4/L5 would increase the coverage of first pelvic recurrences from 42 to 93%, supporting the results from De Bruycker et al. [20].

ADT is accepted as a treatment modality in combination with radiotherapy in primarily diagnosed prostate cancer with regional lymph node metastases. Prospective randomized phase III data from Messing et al. [24, 25] found improved overall survival rates in patients with positive lymph nodes after radical prostatectomy and lymphadenectomy who received immediate ADT (*n* = 47) in comparison to deferred ADT (*n* = 51)—36 vs. 55% of patients died within a median of 11.9 years follow-up. However, as this study included only a small number of patients with a larger number of positive lymph nodes (median 2, range 1–20), ADT is not generally accepted as a treatment for patients with limited lymph node involvement following lymphadenectomy [7].

Patients who received salvage radiotherapy > 15 years ago, especially patients with higher PSA levels, would frequently have been diagnosed with oligometastases with current diagnostic methods. Prospective randomized studies demonstrated a benefit with the combination of salvage radiotherapy and short-term (GETUG-AFU-16, 6 months, improved 10-year progression-free survival of 64 vs. 49%; *p* < 0.01) [26] or long-term (RTOG 9601, 24 months, significantly improved overall survival for patients with presalvage radiotherapy PSA 0.61–1.5 ng/ml, HR 0.61; and > 1.5 ng/ml, HR 0.45) [27, 28] ADT in patients with biochemical recurrence (without PET/CT staging). For patients with metachronous oligometastases in hormone-sensitive prostate cancer, systemic treatment including ADT is the standard of care [29]. However, this concept has recently been challenged in prospective phase II studies, applying local treatment to defer ADT. ADT is not regarded as standard treatment for biochemical recurrence according to current guidelines.

Importantly, local treatment has not yet been compared with the standard of care. In the STOMP trial [13], criteria for initiation of ADT were defined as “symptomatic progression, progression to more than three metastases, or local progression of baseline-detected metastases.” Progression by PSA increase alone was not an indication to start ADT, nor was development of additional metastases amenable to MDT as long as the patient still had three or fewer total metastases. A retrospective multicenter study of 305 PET-positive oligorecurrent prostate cancer patients who received stereotactic or fractionated radiotherapy only showed improved biochemical recurrence-free survival with the addition of ADT for > 6 months after a median follow up of 16 months [30].

In patients with biochemical recurrence or asymptomatic metastases, in particular with long PSA doubling times > 10 months and a longer relapse-free interval > 2 years after initial curative treatment, ADT may be withheld initially without compromising oncologic outcome [31]. For these patients, deferred ADT has the advantage of maintaining quality of life. Patient anxiety, compliance, comorbidities, life-expectancy, and the toxicity of androgen deprivation needs to be taken into account. Intermittent androgen deprivation should be considered, as quality of life can be expected to be better in comparison to continuous androgen deprivation [31]. Patients have to be assessed individually in a multidisciplinary tumor board.

Molecular predictive factors might help to select patients who will particularly benefit from MDT, such as patients with an absence of high-risk mutations in ctDNA. In addition, the effect of radiotherapy on the immune system might induce an in-situ vaccine response [14].

The results of randomized studies in patients with oligorecurrent nodal prostate cancer are eagerly awaited in the next few years, such as the OLIGOPELVIS‑2 trial comparing ADT with ADT+ENRT, and the STORM trial (Salvage Treatment of OligoRecurrent nodal prostate cancer Metastases) comparing salvage lymph node dissection/SABR+ADT with ENRT+ADT.

## Conclusion and recommendations

*Oligorecurrent prostate cancer *is commonly diagnosed following PET/CT staging and defined as a locoregional or distant recurrence in up to three (–five) locations.

### 1. Oligorecurrent prostate cancer with lymph nodes limited to the pelvis

Conventionally fractionated elective *pelvic* nodal radiotherapy with a boost to the involved nodes (simultaneous integrated or sequential boost) should be preferred over involved node SABR only, with lower recurrence rates and possibly improved cancer-specific survival. Standard elective pelvic nodal radiotherapy includes the obturator, presacral, internal, and external iliac lymph nodes. Inclusion of common iliac lymph nodes is also recommended.

### 2. Oligometastatic prostate cancer with distant nodal recurrences

For nodal recurrences outside the pelvis, elective nodal radiotherapy and SABR have not been sufficiently studied. ENRT (specifically paraaortic nodes) or involved-node SABR may be performed in selected cases.

### 3. Androgen deprivation therapy

Systemic treatment should be based on current guidelines, including long-term androgen deprivation therapy as first-line treatment in hormone-sensitive patients. In limited-volume disease, especially if up to three lymph nodes are involved, local treatment could be considered as upfront treatment in individually selected patients, especially in patients with long PSA doubling times > 10 months and longer relapse-free interval > 2 years after initial curative treatment. However, this concept has not yet been prospectively compared with the current standard—radiotherapy combined with androgen deprivation—and should therefore not be used routinely.

We recommend the inclusion of patients with oligorecurrent prostate cancer in prospective clinical studies.
